# Understanding the heterogeneity of pancreatic ductal adenocarcinoma

**DOI:** 10.1016/j.tranon.2025.102479

**Published:** 2025-07-22

**Authors:** Juan Iovanna, Nicolas Fraunhoffer, Raul Urrutia, Nelson Dusetti

**Affiliations:** a Centre de Recherche en Cancérologie de Marseille (CRCM), INSERM U1068, CNRS UMR7258, Aix Marseille Université and Institut Paoli Calmettes, Parc Scientifique et Technologique de Luminy, Equipe labélisée Ligue Nationale contre le cancer, 163 Avenue de Luminy 13288, Marseille, France; b Hospital de Alta Complejidad El Cruce, Florencio Varela, Buenos Aires, Argentina; c University Arturo Jauretche, Florencio Varela, Buenos Aires, Argentina; d Centro de Estudios Farmacológicos y Botánicos (CEFYBO), Facultad de Medicina, Universidad de Buenos Aires-CONICET; e Departamento de Microbiología, Parasitología e Inmunología, Facultad de Medicina, Universidad de Buenos Aires, Buenos Aires, Argentina; f Linda T. and John A. Mellowes Center for Genomic Sciences and Precision Medicine, Medical College of Wisconsin, Milwaukee, Wisconsin, USA; g Department of Surgery, Division of Research, Medical College of Wisconsin, Milwaukee, Wisconsin, USA

**Keywords:** PDAC, Tumor heterogeneity, Metastases, Tumor progression, Tumor microenvironment, Epigenetics

## Abstract

Pancreatic ductal adenocarcinoma (PDAC) is a highly aggressive, treatment-resistant cancer characterized by extensive inter- and intra-tumoral heterogeneity. Although over 95 % of cases harbor KRAS mutations and commonly altered tumor suppressors like TP53, SMAD4, and CDKN2A, these genetic changes alone do not fully explain PDAC variability. We propose a paradigm shift: PDAC heterogeneity is not solely genetic but also shaped by epigenetic regulation and the tumor microenvironment. Traditional transcriptomic classifications define PDAC into fixed subtypes, primarily classical and basal-like, but we argue these are not static categories. Instead, PDAC phenotypes exist along a dynamic continuum influenced by stromal interactions and epigenetic cues. This model challenges the binary classification view. We show that transitions from classical to basal-like states are gradual and reversible, driven by tumor-stroma crosstalk and chromatin remodeling. Such plasticity underpins tumor adaptation, resistance, and progression. Embracing this dynamic framework offers novel therapeutic opportunities.

## Highlights

• **PDAC is highly heterogeneous and treatment-resistant**, with tumor variability that cannot be fully explained by common genetic mutations such as *KRAS, TP53, SMAD4*, and *CDKN2A*.
• **A paradigm shift is proposed**: PDAC heterogeneity is driven not only by genetics but also by **epigenetic regulation and the tumor microenvironment**.
• **Traditional transcriptomic classifications (classical vs. basal-like) are inadequate**, as PDAC phenotypes exist along a **dynamic and reversible continuum**, rather than fixed subtypes.
• **Transitions between classical and basal-like states are gradual**, regulated by **tumor-stroma crosstalk and chromatin remodeling**, highlighting the plasticity of PDAC.
• **This dynamic framework opens new therapeutic opportunities** by targeting tumor plasticity and microenvironmental interactions to overcome treatment resistance.

## Graphical abstract

**Figure:**
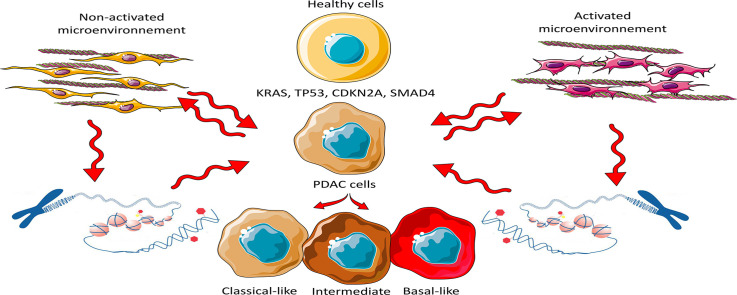

## Introduction

Pancreatic ductal adenocarcinoma (PDAC) is one of the most aggressive malignancies, characterized by its high resistance to available treatments and a generally poor prognosis [1]. One of the main challenges in its clinical and therapeutic management is its marked heterogeneity among patients and within the same tumor. Although approximately 95 % of PDAC cases harbor KRAS mutations, alterations in tumor suppressor genes vary significantly. TP53 is mutated in 50-75 % of cases, SMAD4 is inactivated in about 55 %, and CDKN2A is altered in nearly 90 % [1]. However, this genetic diversity does not directly correlate with distinct tumor phenotypes [2,3]. This suggests that other factors, such as epigenetic regulation and the tumor microenvironment, play a crucial role in PDAC behavior and progression. Transcriptomic studies have enabled the classification of PDAC into different molecular subgroups, each associated with variations in aggressiveness, chemotherapy response, and patient survival [[3], [4], [5], [6], [7]]. This classification has improved our understanding of tumor biology and opened the door to more personalized therapeutic strategies. Within a single tumor, a high degree of cellular diversity is often observed [[8], [9], [10], [11]]. In addition to tumor cells, the PDAC microenvironment includes cancer-associated fibroblasts (CAF), immune cells, endothelial cells, and a dense extracellular matrix [[9], [10], [11]]. This complex cellular network creates a dynamic environment that promotes tumor plasticity, allowing the coexistence of multiple subclones with different proliferation, invasion, and treatment resistance capacities. The presence of heterogeneous cellular subpopulations within the tumor drives the natural selection of clones that are more adapted to therapeutic pressures [12,13]. As the disease progresses, resistant clones may emerge, facilitating tumor progression and increasing the risk of relapse after therapy. This inter- and intra-tumoral heterogeneity translates into high variability in treatment response [14,15]. Patients with highly heterogeneous tumors tend to experience a more aggressive clinical course, with a greater likelihood of chemotherapy resistance and, consequently, a worse prognosis. Conversely, some subtypes with lower heterogeneity or favorable molecular characteristics have shown better therapeutic responses, emphasizing the importance of personalized medicine in PDAC treatment. The heterogeneity of PDAC represents a fundamental challenge in both research and clinical practice. Understanding the mechanisms behind this heterogeneity is crucial for developing new therapeutic strategies that can improve treatment efficacy and patient outcomes. One key question remains whether the tumor arises and maintains a stable phenotype or progressively evolves into more aggressive forms over time.

## Histopathological classifications reflect the existence of PDAC subtypes

From a histological perspective, PDAC can present in various forms, each with specific characteristics that influence its prognosis and response to treatment: i/ Conventional, the most common form, with disorganized glandular architecture and a high degree of desmoplasia; ii/ Adenosquamous, contains both glandular and squamous components, associated with a worse prognosis; iii/ Colloid (Mucinous), characterized by the abundant production of extracellular mucin, generally linked to a better prognosis; iv/ Medullary, poorly differentiated tumors with strong lymphocytic infiltration, frequently associated with microsatellite instability; and v/ Giant Cell, a rare variant with multinucleated tumor cells, whose biology is not yet fully understood [16].

## The Birth of PDAC molecular subtypes via transcriptomics

Transcriptome analysis enabled the classification of PDAC into molecular subtypes, a methodology previously applied to other cancers, such as lymphoma and breast cancer. Although approaches and materials have varied across studies, two major PDAC lineages have been identified with multiple proposed variations. One of the first studies in this field was conducted by Collisson et al. (2011) [4], who analyzed untreated resected primary tumors. Using micro dissected epithelium, they defined three subtypes: i/ Classical, characterized by high GATA6 expression and strong KRAS dependency; ii/ Quasi-Mesenchymal (QM-PDA), more aggressive, associated with a worse prognosis and higher tumor grade; and iii/ Exocrine-like, a less characterized subtype with lower clinical relevance. In 2015, Moffitt et al [5] expanded these findings by analyzing both primary and metastatic tumors, they removed transcripts derived from normal pancreatic tissue and the tumor microenvironment to minimize interference from high stromal content in genomic analyses. This allowed them to identify i/ two tumor subtypes, basal-like and classical, and ii/ two stromal subtypes, normal and activated. Later, in 2016, Bailey et al [3] analyzed 266 resected primary tumors with high epithelial cellularity using RNA-seq, defining four main subtypes: i/ Squamous, highly aggressive and associated with a poor prognosis; ii/ Pancreatic Progenitor, characterized by a gene expression profile similar to pancreatic progenitor cells; iii/ Immunogenic, distinguished by a strong presence of immune infiltrate-derived transcripts; and iv/ ADEX (Aberrantly Differentiated Endocrine-Exocrine), linked to exocrine and endocrine differentiation characteristics. The immunogenic subtype was a novel finding, suggesting a higher immune response in some PDAC cases, which could influence the efficacy of immunotherapy treatments. In 2018, Puleo et al [6] redefined PDAC subtypes using formalin-fixed, paraffin-embedded samples, making classification more applicable in clinical settings. Through the analysis of 309 samples, they used advanced gene expression and targeted sequencing techniques to identify five distinct subtypes: i/ Pure Basal-like, similar to the Squamous subtype, associated with worse prognosis; ii/ Stroma Activated, enriched in stromal signals, which may affect response to anti-fibrotic therapies; iii/ Desmoplastic, characterized by strong interaction with the tumor microenvironment; iv/ Pure Classical, more differentiated tumors with a better prognosis; and v/ Immune Classical, shows strong immune system interaction, with potential for immunomodulatory therapies. In 2020, Nicolle et al [7] proposed a new approach, describing PDAC as a continuous gradient rather than discrete subtypes. To achieve this, they analyzed five histological groups and constructed a molecular gradient, PAMG, based on tumor transcriptomics. This gradient reflects a progression from a Classical to a Basal phenotype, with implications for tumor evolution and treatment response. The PAMG model was validated in independent cohorts of resected, advanced, and metastatic tumors, demonstrating independent prognostic value and improving the characterization of PDAC heterogeneity compared to previous classifications. This model challenges the classical/basal dichotomy, proposing a more dynamic system that better reflects tumor evolution over time. To support the conceptual foundation of the proposed continuum model, we underscore the existence of a dynamic phenotypic spectrum that transcends the traditional binary classification of PDAC into classical and basal subtypes. Rather than representing fixed and mutually exclusive states, tumor cell phenotypes are increasingly understood as reversible and fluid, shaped by epigenetic reprogramming and microenvironmental cues, particularly from the stroma. This phenotypic plasticity reflects an adaptive response to environmental stressors such as hypoxia, inflammation, and nutrient deprivation. Recent single-cell transcriptomic analyses further reinforce this model, revealing the presence of hybrid subpopulations that simultaneously express features of both classical and basal phenotypes, highlighting the continuum as a biologically relevant framework [8,17].

Altogether, these studies have allowed for a deeper understanding that PDAC classification is not strictly binary but exists within a continuous spectrum of differentiation, where tumors may evolve from a more differentiated (Classical) to a more aggressive (Basal) state. The Squamous, QM-PDAC, and Basal-like subtypes correlate with worse prognosis, whereas the Classical subtype remains consistent across classifications as a less aggressive tumor form. Additionally, this Classical subtype presents key markers such as HNF4A and GATA6, which could be useful for developing targeted therapies. Shifting from a binary classification to a gradual and dynamic model allows a better understanding of PDAC's biological and clinical heterogeneity. This new approach carries critical therapeutic implications, as it enables the identification of potential therapeutic targets, predicts treatment responses, and facilitates the development of more personalized treatment strategies for each patient. Ultimately, PDAC classification continues to evolve, enhancing our ability to design more effective and personalized therapies for this devastating disease.

## PDAC classification in the era of system biology

PDAC is a disease with high biological heterogeneity, leading to the development of various classification systems based on genomic, microenvironmental, and therapeutic response profiles. These classification systems enhance our understanding of tumor biology and provide opportunities for more precise and personalized therapeutic approaches.

## Genomic subtypes of PDAC inform precision therapeutics

At the genetic level, PDAC presents recurrent mutations in key genes that are mandatory for its development but with a lesser influence on its progression: i/ Mutations in KRAS, TP53, CDKN2A, and SMAD4, these alterations are present in most PDAC cases but do not strictly determine the tumor phenotype; ii/ Microsatellite instability-high (MSI-high) subtype, rare in PDAC, but represents a group of tumors with potential sensitivity to immune checkpoint inhibitors, and iii/ DNA repair deficiency subtype (mutations in BRCA1/BRCA2): Tumors in this category show sensitivity to PARP inhibitors and platinum-based chemotherapy, suggesting a specific therapeutic opportunity [1,18].

## PDAC stromal subtypes impact tumor evolution and treatment responses

PDAC is defined by its tumor cells and interactions with its microenvironment, which influence its evolution and response to treatment. Three main tumor stroma categories have been identified based on composition and activity: i/ Activated stroma, composed of CAF with high extracellular matrix production, which promotes treatment resistance and hinders drug penetration; ii/ Inflammatory stroma, characterized by more significant immune cell infiltration, which may increase sensitivity to immunotherapy-based treatments; and iii/ Inactive stroma, defined by less fibrosis and lower tumor aggressiveness, suggesting a better response to specific conventional treatments [19].

## PDAC subtypes differ in chemosensitivity, immunotherapeutic potential, and DNA repair targeting response

PDAC classification can also be based on treatment response, which has direct implications in the selection of therapeutic strategies: i/ Tumors sensitive to conventional chemotherapy, Classical subtypes tend to respond better to gemcitabine/nab-paclitaxel-based regimens; ii/ Chemoresistant tumors, tumors with a basal phenotype are more resistant to fluoropyrimidines and platinum-based therapies, limiting the efficacy of standard treatments [20]; iii/ Candidates for immunotherapy: tumors with microsatellite instability (MSI-high) or a microenvironment rich in immune infiltration may benefit from immune checkpoint inhibitors [21]; and iv/ Tumors sensitive to PARP inhibitors, those with BRCA1/2 mutations or DNA repair defects have shown favorable responses to PARP inhibitors [22], representing a promising therapeutic strategy for this subgroup.

## Two hypotheses to explain the PDAC heterogeneity

Combined, these classifications demonstrate a significant degree of tumor heterogeneity. We sought to address how this heterogeneity occurs. When analyzing the PDAC phenotype at a specific moment, what is observed is essentially a “snapshot” of the tumor at that phase of its evolution. However, this does not clarify whether the phenotype remains stable or evolves into a more aggressive form. Notably, two hypotheses have been proposed to explain this heterogeneity: i/ Phenotypic Stability Hypothesis (Possibility 1), the tumor phenotype is defined from the initial cellular transformation and remains unchanged throughout the disease course; and ii/ Phenotypic Plasticity Hypothesis (Possibility 2), the tumor phenotype progressively evolves, influenced by internal and external factors, such as the tumor microenvironment, cellular stress, and epigenetic interactions. Thus, in the following paragraphs, we will explore the biological determinant of PDAC heterogeneity.

## Tumor plasticity in PDAC as a candidate phenomenon to explain PDAC heterogeneity that gives rise to tumor subtypes

**Fig. 1:**
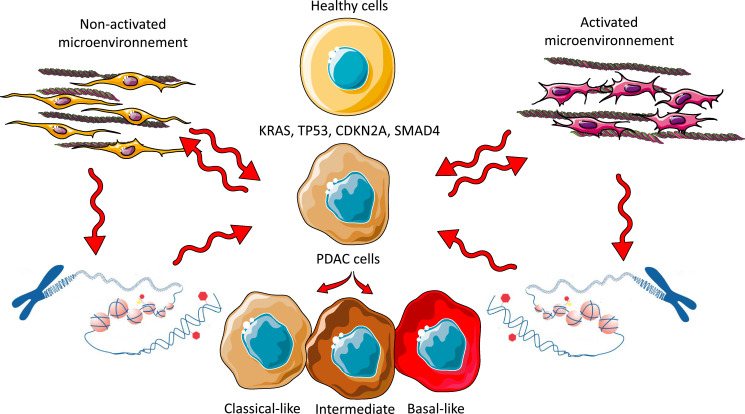
Cell phenotype is determined by the complex interaction between the tumor microenvironment and the Epigenetic Regulation of Epithelial Cells rather than by the Accumulation of Genetic Mutations. The schematic representation illustrates how mutations in driver genes can trigger the malignant transformation of pancreatic cells without directly influencing their phenotype. In contrast, the stroma plays a crucial role in phenotype determination through direct interactions with tumor cells or by modulating epigenetics, thereby influencing cellular plasticity and tumor progression.

PDAC represents a dynamic system in which distinct phenotypic states coexist [12]. The coexistence of classical and basal phenotypes within the same tumor suggests that PDAC is not composed of fixed entities but rather a dynamic spectrum of cellular states. This hypothesis is reinforced by experiments in pancreatic organoids, where cells derived from the same source can give rise to subpopulations with markers of both phenotypes [8]. Moreover, the analysis of 45,293 tumor cells from the Werba et al [23] single-cell cohort, scored using the classical and basal-like markers identified by Raghavan et al [24] and the master regulator gradient (PAMGMR) [25] reveals that the cells are distributed along a phenotypic continuum, with some exhibiting a hybrid/intermediate state. Chan-Seng et al. reported similar results [17] where they refer to the hybrid phenotype. Single-cell RNA sequencing studies have revealed that basal cells may retain classical phenotype transcripts and vice versa [8], indicating that tumor cells do not entirely abandon one phenotypic program before acquiring another but can exist in an intermediate state. Table 1 presents a comprehensive evaluation of the evidence supporting PDAC plasticity. To further consolidate the proposed model of phenotypic plasticity in PDAC, we have integrated mechanistic evidence into our conceptual framework. For instance, Juiz et al [8] and Chan-Seng-Yue et al [17] have demonstrated the coexistence of classical and basal-like phenotypes within individual tumors by employing organoid cultures and single-cell transcriptomic analyses. These studies reveal the presence of hybrid cellular states that challenge the notion of rigid subtype boundaries. Their findings support a model in which tumor phenotypes exist along a continuum, shaped by microenvironmental cues and epigenetic regulation, rather than being strictly determined by genetic alterations alone (Fig. 1).

**Table 1:** Conceptual framework of PDAC phenotypic evolution and heterogeneity.

**1. Coexistence of Classical and Basal Phenotypes within the Same Tumor** PDAC is not composed of fixed cellular entities but rather represents a dynamic system in which distinct phenotypic states coexist. The presence of both classical and basal phenotypes within a single tumor suggests that tumor cells retain a degree of plasticity, enabling transitions between phenotypic states rather than adhering to a rigid, predetermined trajectory [8,11,12,24,49].
**2. Divergence of Tumor Subpopulations from a Common Cell of Origin** A single population of tumor-initiating cells can give rise to distinct subpopulations, each acquiring molecular features characteristic of both classical and basal phenotypes. This process is likely influenced by microenvironmental cues and regulatory mechanisms that modulate cellular identity [11,17,24,50,51].
**3. Common Oncogenic Mutations in Classical and Basal Phenotype Tumors** Canonical PDAC driver mutations (*KRAS, TP53, CDKN2A, SMAD4*) are shared between classical and basal subtypes, indicating that genetic alterations alone do not dictate phenotypic divergence. Instead, post-transcriptional modifications, epigenetic regulation, and tumor-stroma interactions appear to be the principal determinants of phenotypic identity and tumor progression [6,17,24].
**4. Retention of Classical Phenotype Transcripts in Basal Cells and Vice Versa** Single-cell RNA sequencing studies have demonstrated that basal-like cells may retain transcripts associated with the classical phenotype, and vice versa. This finding suggests that phenotypic transitions in PDAC are not absolute but instead occur through intermediate states where molecular signatures of both phenotypes coexist. Such plasticity supports the concept of PDAC as a continuous spectrum of cellular states rather than a dichotomous classification [6,17,24].
**6. Differential Stroma Activation in Classical and Basal Subtypes** The tumor-associated stroma contributes to PDAC heterogeneity by modulating tumor cell behavior. The classical subtype is generally associated with a less reactive, non-activated stromal microenvironment, whereas the basal subtype is linked to an activated stroma characterized by increased inflammatory signaling and extracellular matrix remodeling. This differential stromal activation may facilitate invasion and metastasis in the basal subtype[5,6,47,51,52].
**7. Epigenetic Regulation and the Influence of the Tumor Microenvironment** PDAC phenotypes are not strictly determined by genetic mutations but are substantially influenced by epigenetic modifications governed by the tumor microenvironment. Epigenetic regulators dynamically respond to external stimuli, modulating gene expression and enabling tumor cells to transition between phenotypic states in response to selective pressures [24,49,53].
**8. Phenotypic Evolution from Early-Stage to Metastatic PDAC** Clinical observations indicate that early-stage, operable tumors predominantly exhibit a classical phenotype, whereas metastatic PDAC is more frequently characterized by a basal, undifferentiated phenotype. This shift supports the hypothesis that phenotypic evolution occurs throughout disease progression, with selective pressures driving the emergence of more aggressive basal-like subpopulations. These findings underscore the importance of early intervention to prevent the transition to a highly invasive phenotype [11,54,55].
**9. Influence of Cellular Origin on Initial Tumor Development** The cellular origin of PDAC influences early tumorigenic processes but does not strictly determine later disease progression. Ductal cells exhibit a high predisposition to forming carcinoma *in situ*, rapidly progressing to invasive PDAC, whereas acinar-derived tumors require an intermediate acinar-to-ductal metaplastic (ADM) transition. However, these initial differences may be overshadowed by epigenetic and microenvironmental factors that contribute to phenotypic convergence in advanced disease stages [[56], [57], [58], [59]].

**Fig. 2:**
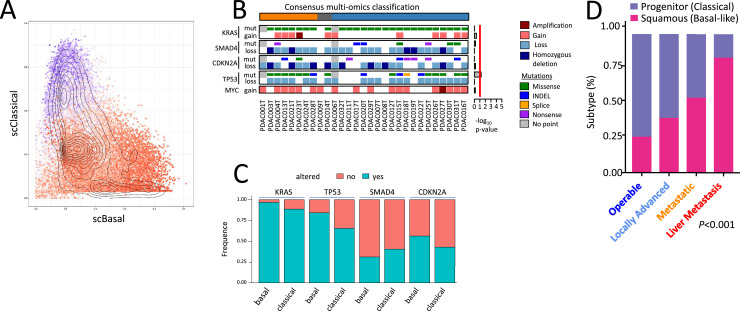
A. Single-cell analysis reveals basal, classical, and chimeric phenotypes within the same PDAC Tumor. Single-cell RNA sequencing (scRNA-seq) analysis of PDAC samples reveals the coexistence of basal, classical, and chimeric phenotypes within the same tumor. B. Genome-Wide Mutational Analysis Indicates no Significant Correlation Between Specific Genetic Mutations and PDAC Phenotypes. The mutational landscape of key driver genes KRAS, TP53, SMAD4, and CDKN2A was examined across 39 patient-derived xenografts (PDXs) previously classified as Basal or Classical. Lateral bars represent the -log10 p-value from Fisher’s exact test, assessing the association between mutations and the consensus multiomics classification. Statistically significant associations (p ≤ 0.05, indicated by a red threshold line) are highlighted, with bars colored orange for basal and blue for classical subtypes. C. Proportion of Each Mutant Analyzed on the TGAC cohort. No significant association was found between mutations with the phenotype of the PDAC. D. Distribution of PDAC Subtypes Across Disease Stages. The proportion of Basal and Classical PDAC subtypes was assessed through transcriptomic analysis of samples from different disease stages, including Operable (n=35), Locally Advanced (n=244), Metastatic (n=76), and Liver Metastases (n=88). This classification provides insights into the distribution of molecular subtypes across the clinical spectrum of PDAC.

Additionally, previous studies have shown that widely reported genetic alterations in PDAC, such as mutations in KRAS and TP53, losses of SMAD4, CDKN2A inactivation, and MYC amplification, are common in both subtypes without a specific association with either [1,2]. Even gene fusion events, which could act as potential tumor drivers, have not shown any preferential relationship with the basal or classical phenotype. Similarly, chromosomal instability indices (CIN) and mutation rates have not demonstrated any significant correlation with the molecular classification of PDAC. One of the few genes showing a slight difference between the subtypes is TP53, with a higher mutation rate in the basal subtype, although with insufficient discriminative power [1,2] (Fig. 2). In addition, we analyzed the frequency of these mutations in the TACG cohort and confirmed the absence of a significative association between mutations and phenotype [3] (Fig. 2). This suggests that the differentiation between the basal and classical phenotypes is not genetically determined but it results from epigenetic and transcriptional regulatory mechanisms. In conclusion, current evidence suggests that specific combinations of recurrent mutations do not determine the basal and classical subtypes of PDAC but rather emerge as epigenetically regulated entities.

## Integrative dynamics of tumor microenvironment in shaping PDAC heterogeneity

The tumor microenvironment is another key factor in the evolution of PDAC. Elements such as hypoxia [26], inflammation [27], and extracellular matrix [28] interactions can promote the selection of cells with more aggressive characteristics. Experimental studies have demonstrated that adverse conditions, such as oxidative stress [29] and exposure to inflammatory cytokines [30], can activate signaling pathways that drive the transition toward a more invasive basal phenotype. Since PDAC heterogeneity cannot be explained solely by genetic alterations, it results from a dynamic balance between epigenetic regulation, microenvironmental interactions, and certainly by the therapeutic pressures. The traditional binary classification model (classical vs. basal) is replaced by a more integrative perspective, in which PDAC behaves as an evolving system. Phenotypic plasticity suggests that therapeutic strategies targeting the epigenome and reprogramming the tumor microenvironment could be crucial for enhancing treatment response and preventing progression toward more aggressive states. The role of the microenvironment is supported by the fact that classical and basal subtypes are associated with non-activated or activated stroma, respectively, in an interactive molecular dialogue. Ultimately, developing more effective therapies will depend on our ability to understand and modulate the mechanisms driving PDAC heterogeneity, enabling personalized strategies to improve patient prognosis. In this context, several clinical studies have explored stroma-targeting strategies. Notably, defactinib, a focal adhesion kinase (FAK) inhibitor [31], and PEGPH20, an enzyme targeting hyaluronan in the extracellular matrix (ECM) [32], have been evaluated in clinical trials. Although PEGPH20 initially enhanced drug delivery in early-phase studies, it failed to demonstrate survival benefits in phase III trials. These results highlight the biological complexity of the tumor stroma and underscore the necessity of more nuanced therapeutic approaches that take into account the heterogeneity and adaptability of the stromal compartment.

## Epigenetic reprogramming drives PDAC heterogeneity

Epigenetic reprogramming is emerging as a fundamental mechanism in PDAC evolution, regulating the expression of master transcription factors and signaling pathways involved in cell differentiation and treatment resistance [33]. Unlike genetic mutations, which are irreversible changes in the DNA sequence, epigenetic modifications are dynamic and reversible, suggesting that they could be therapeutically modulated to alter tumor behavior. In 2018, Lomberk et al [34] the most relevant epigenetic markers were analyzed in dozens of PDAC samples with distinct phenotypes. This study represented the most comprehensive analysis to date on epigenomic landscapes underlying PDAC heterogeneity. The data obtained allowed the characterization of transcriptional processes regulated by promoters, enhancers, and super-enhancers in classical and basal PDAC subtypes. One of the most significant findings was the influence of environmental factors and the tumor microenvironment in shaping these epigenetic landscapes. It was observed that these landscapes do not spontaneously interconvert, reinforcing the idea that tumor subtypes can remain differentiated over time. However, inhibiting key regulatory pathways, such as MET in super-enhancers, demonstrated that phenotypic plasticity is a real and potentially modulable phenomenon. This finding suggests that specific therapeutic agents could alter tumor aggressiveness, opening new opportunities for targeted strategy development. Experimental models, supporting the exploration of anti-MET therapies, currently in clinical trials for other cancers. Additionally, data on DNA methylation and histone modifications further reinforce the feasibility of developing epigenetic therapies to modify chromatin structure and regulate gene expression.

Key transcription factors, such as ZEB1, SNAIL, and TWIST, have been widely implicated in the epithelial-mesenchymal transition (EMT), a critical process in the progression of PDAC to a more aggressive phenotype [35]. This transition enhances migration and invasion and contributes to treatment resistance. Furthermore, changes in chromatin accessibility have been identified in promoter regions of genes associated with the basal phenotype, suggesting that the structural remodeling of the genome directly influences tumor plasticity.

## The cellular origin significantly influences PDAC phenotype in mice

Murine GEMM models of PDAC induced by KRAS mutations and TP53 inactivation in both acinar and ductal cells suggest that the cellular origin of oncogenic mutations significantly influences PDAC phenotype. A 2019 study by Lee et al [36] reported that transformed ductal cells are highly predisposed to forming carcinoma *in situ*, rapidly progressing to invasive PDAC, whereas acinar-derived PDAC requires an intermediate metaplastic transition. However, these findings lack solid clinical validation. Extrapolating murine models to human disease is problematic, as the pancreatic microenvironment in patients is far more complex, shaped by systemic factors that influence tumor progression. Moreover, clinical studies have not established a correlation between cellular origin and human PDAC aggressiveness. Tumor heterogeneity appears to be driven more by cellular plasticity than by the initial cell type harboring mutations. Furthermore, the assumption that ductal cells require fewer additional alterations for malignancy oversimplifies a highly dynamic process. Evidence from human studies suggests that chronic inflammation and epigenetic reprogramming play a more decisive role in malignant transformation than the cellular origin itself. In conclusion, while murine models provide valuable mechanistic insights, their clinical relevance remains uncertain. To validate these observations in the human context, broader studies incorporating environmental, immunological, and genetic factors are necessary before considering therapeutic strategies based on the cell of origin.

## Evolution from classical to basal phenotype and its impact on PDAC progression

A recent study conducted in our laboratory analyzed the transcriptome of 443 patients, stratified into three categories: 35 with operable tumors, 244 with locally advanced tumors, and 164 with metastatic disease. Among the metastatic cases, 76 samples were from primary tumors, and 88 were from liver metastases. We examined their phenotype by using the Bailey et al. method [3]. Then we analyzed the distribution of classical and basal-like phenotypes according to different PDAC clinical stages and found a progressive increase in the basal-like phenotype: 24 % in operable disease, 37 % in locally advanced tumors, and 51 % and 82 % in primary metastatic tumors and liver metastases, respectively (p< 0.001). If tumor phenotype were strictly determined by its origin (phenotypic stability hypothesis), its distribution would remain constant across disease stages. However, our findings reveal that early-stage tumors are predominantly classical, whereas most metastatic tumors exhibit a basal-like phenotype. This strongly suggests that PDAC evolves into a more aggressive phenotype as the disease advances. Our results support the hypothesis that epigenetic and transcriptomic plasticity, rather than the accumulation of additional genetic mutations, primarily drives this transformation. Consequently, PDAC should not be viewed as a disease with fixed subtypes but rather as a dynamic continuum in which tumor cells progressively shift between phenotypic states in response to environmental changes.

## Unlocking new frontiers for personalized therapy

Understanding epigenetic reprogramming in PDAC has opened new opportunities for developing more effective personalized therapies. Since epigenetic mechanisms strongly influence tumor plasticity and PDAC progression, various strategies have been proposed to modulate these processes and improve treatment responses [35]. One of the main approaches is epigenetic modulation to revert the basal phenotype, which is associated with greater aggressiveness and resistance to conventional therapies. In this context, drugs targeting epigenetic enzymes responsible for DNA methylation and histone modifications could induce a partial reversion to the classical phenotype, increasing tumor sensitivity to chemotherapy. Furthermore, it has been demonstrated that inhibitors of key super-enhancers, such as MET, can alter tumor phenotypic identity, reducing aggressiveness and metastatic potential [34]. Another key strategy focuses on modulating tumor plasticity, as PDAC phenotypic transition appears to be driven mainly by the tumor microenvironment. In this regard, the design of therapies aimed at blocking tumor stroma signals or reprogramming CAF could prevent tumor evolution toward a more aggressive basal phenotype [37]. Likewise, agents capable of modifying chromatin accessibility and regulating transcription factor activity could disrupt the EMT, a process essential for PDAC metastatic dissemination. Moreover, a promising strategy is the combination of epigenetic therapies with immunotherapy. Certain tumors with specific epigenetic profiles may respond better to immune checkpoint inhibitors if gene expression patterns are previously modified through epigenetic reprogramming. This suggests that epigenetic modulation could enhance tumor immunogenicity, thereby increasing immunotherapy efficacy and improving the immune system’s response against tumor cells.

To reinforce the translational relevance of tumor plasticity, we highlight emerging therapeutic strategies aimed at epigenetic reprogramming. Histone deacetylase (HDAC) inhibitors, such as vorinostat and romidepsin [38], and bromodomain and extraterminal domain (BET) inhibitors like birabresib (OTX015) [39], are currently undergoing early-phase clinical evaluation for their capacity to remodel chromatin architecture and alter gene expression profiles. These agents hold the potential to reprogram basal-like tumor phenotypes toward a more differentiated, treatment-responsive classical state. By modulating the epigenetic landscape, such approaches may enhance the efficacy of conventional chemotherapies and immunotherapies in patients with PDAC.

Collectively, these strategies provide an innovative approach to controlling PDAC progression by targeting tumor cells in their current state and modulating phenotypic plasticity to prevent the selection of more aggressive subpopulations. Integrating epigenetic therapies with immunotherapy and conventional treatments could represent a paradigm shift in PDAC management, enabling better treatment responses and reduced tumor resistance, ultimately improving patient outcomes.

## Integrative mechanisms driving the phenotypic continuum in PDAC

Recent advances in transcriptomic profiling and organoid modeling have revealed that PDAC is not accurately defined by a binary classification of classical versus basal-like subtypes [7]. Instead, PDAC should be conceptualized as a dynamic phenotypic continuum, wherein tumor cells exhibit varying degrees of epithelial or mesenchymal features, often co-existing within the same lesion and fluctuating in response to environmental pressures. This phenotypic plasticity is governed not only by genetic alterations, but also, and overall, by the epigenetic reprogramming, which acts as a master regulator of state transitions [34,35].

## TGF-β signaling as a driver of plasticity

**Fig. 3:**
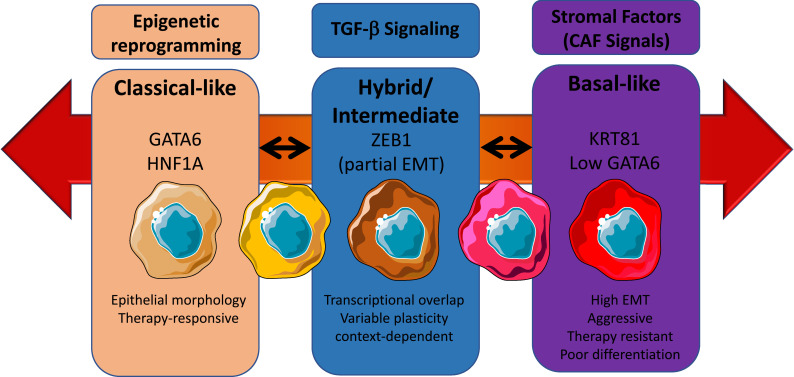
Phenotypic continuum model of PDAC. This schematic represents a dynamic, non-binary model of PDAC cellular states, emphasizing phenotypic plasticity across a spectrum from classical to basal-like subtypes. In contrast to traditional classifications that treat subtypes as mutually exclusive, this continuum highlights reversible and transitional cellular identities, influenced by epigenetic reprogramming, TGF-β signaling, and stromal-derived cues such as those from cancer-associated fibroblasts (CAFs). On the left, the classical phenotype is characterized by epithelial morphology, strong expression of GATA6 and HNF1A, and greater responsiveness to conventional therapies. In the center, an intermediate or hybrid state emerges, marked by partial EMT (epithelial–mesenchymal transition), expression of S100A2 and ZEB1, and a context-dependent transcriptional program. These cells exhibit high plasticity, enabling them to shift toward either end of the spectrum depending on environmental pressures. On the right, the basal-like phenotype exhibits mesenchymal morphology, expression of markers such as KRT81, TP63, and low GATA6, along with features of high EMT, invasiveness, and resistance to therapy. Bidirectional arrows between these states indicate that phenotypic transitions are reversible, reflecting the underlying plasticity of PDAC cells. This flexibility poses a major challenge for therapeutic targeting, as tumor cells can dynamically adapt to selective pressures, including treatment and microenvironmental changes.

Transforming growth factor beta (TGF-β) is abundantly secreted in the PDAC tumor microenvironment and plays a dual role in disease progression. While initially acting as a tumor suppressor in early lesions, in advanced PDAC TGF-β promotes epithelial-to-mesenchymal transition (EMT), immune evasion, and metastasis [40]. Mechanistically, TGF-β induces expression of transcription factors such as ZEB1, SNAIL, and TWIST, which repress epithelial genes and activate mesenchymal programs [41,42]. Importantly, ZEB1 can recruit epigenetic modifiers, including histone deacetylases (HDACs) and components of the Polycomb repressive complex 2 (PRC2), leading to chromatin remodeling and durable transcriptional repression [43]. Furthermore, TGF-β has been shown to upregulate KDM2B, a histone[44] demethylase that contributes to stable epigenetic silencing of epithelial lineage genes, reinforcing the basal-like phenotype. These mechanisms contribute to a shift in transcriptional identity, enabling tumor cells to adapt dynamically to stressors and progress toward more aggressive, poorly differentiated states (Fig. 3).

## Epigenetic reprogramming and reversibility of state transitions

One of the defining features of PDAC plasticity is its epigenetic flexibility, allowing transitions between phenotypic states without permanent genetic alterations. Classical PDAC tumors are characterized by open chromatin at loci encoding differentiation-promoting transcription factors, such as GATA6 and HNF1A, while basal-like tumors exhibit repressive chromatin at these loci and activate inflammatory and stress-related programs [45]. These chromatin landscapes are malleable and can be reshaped by environmental cues, such as hypoxia, nutrient deprivation, and therapeutic pressure. Notably, pharmacologic inhibition of HDACs and bromodomain and extraterminal domain proteins has been shown to partially reverse the mesenchymal phenotype, reactivating classical gene expression and restoring sensitivity to chemotherapy and immunotherapy [46]. This suggests that targeting the epigenetic machinery may offer a route to modulate cell identity and overcome resistance.

## Microenvironmental influences and stromal crosstalk

The tumor microenvironment plays a fundamental role in maintaining phenotypic heterogeneity in PDAC. Among its most influential components are cancer-associated fibroblasts (CAFs), which exist in multiple functionally distinct subtypes, including myofibroblastic CAFs (myCAFs), inflammatory CAFs (iCAFs), and antigen-presenting CAFs (apCAFs) [47]. These cells produce a diverse array of signaling molecules, such as TGF-β, IL-6, and CXCL12, as well as extracellular matrix components that modulate tumor cell behavior. CAFs influence tumor cell plasticity through paracrine loops that sustain EMT and mesenchymal programming. Additionally, mechanotransduction signals from ECM stiffness can impact nuclear architecture and chromatin accessibility, further enforcing mesenchymal transcriptional states [48]. Thus, the stroma is not merely a passive scaffold but an active regulator of epigenetic and transcriptional identity in PDAC cells (Fig. 3).

## Clinical relevance of the phenotypic continuum

The existence of a phenotypic spectrum in PDAC has profound implications for therapy. Tumors composed of a mixture of classical and basal-like cells, or those in an intermediate/hybrid state, may respond unpredictably to standard treatments. The plastic nature of these tumors allows for rapid adaptation to therapeutic stress through state switching. Consequently, therapeutic strategies must go beyond targeting fixed molecular subtypes and instead address the mechanisms that drive plasticity itself. This could involve combinations of epigenetic therapies (e.g., HDAC or BET inhibitors), TGF-β pathway blockade, and stroma-modulating agents, selected based on a tumor’s position within the phenotypic spectrum. Such adaptive approaches will be essential to outpace the evolving biology of this highly lethal cancer.

## Concluding remarks

PDAC is not a static disease; instead, it exhibits a continuum of phenotypic transition, driven more by epigenetic and transcriptomic regulation than by additional genetic alterations. This tumor plasticity enables PDAC to evolve into more aggressive forms over time, which has direct implications for classification and treatment. Targeted therapies should not focus solely on attacking tumor cells at a specific phenotypic state. Still, they should also consider modulating tumor plasticity to prevent the selection of more aggressive and resistant subpopulations. Epigenetic modulation emerges as a promising strategy to reverse the basal phenotype or prevent its emergence, enhancing responses to conventional treatments and reducing tumor progression. In the future, the combination of epigenetic, targeted, and immunotherapy treatments could represent a more effective approach to PDAC management, offering new opportunities to improve patient survival.

## CRediT authorship contribution statement

**Juan Iovanna:** Writing – original draft, Supervision, Investigation, Conceptualization. **Nicolas Fraunhoffer:** Writing – original draft, Conceptualization. **Raul Urrutia:** Writing – original draft, Conceptualization. **Nelson Dusetti:** Writing – original draft.

## Declaration of competing interest

The authors disclose no conflicts of interest.
